# Androgens Induce Invasiveness of Triple Negative Breast Cancer Cells Through AR/Src/PI3-K Complex Assembly

**DOI:** 10.1038/s41598-019-41016-4

**Published:** 2019-03-14

**Authors:** Pia Giovannelli, Marzia Di Donato, Ferdinando Auricchio, Gabriella Castoria, Antimo Migliaccio

**Affiliations:** Department of Precision Medicine, University of Campania ‘L. Vanvitelli’, Via L. De Crecchio, 7, 80138 Naples, Italy

## Abstract

Breast cancer (BC) is still characterized by high morbidity and mortality. A specific BC subtype named triple negative BC (TNBC) lacks estrogen and progesterone receptors (ER and PR, respectively) and is characterized by the absence of overexpression/amplification of human epidermal growth factor receptor 2 (HER2). The androgen receptor (AR) is expressed in TNBC, although its function in these cancers is still debated. Moreover, few therapeutic options are currently available for the treatment of TNBC. In this study, we have used TNBC-derived MDA-MB231 and MDA-MB453 cells that, albeit at different extent, both express AR. Androgen challenging induces migration and invasiveness of these cells. Use of the anti-androgen bicalutamide or AR knockdown experiments show that these effects depend on AR. Furthermore, the small peptide, S1, which mimics the AR proline-rich motif responsible for the interaction of AR with SH3-Src, reverses the effects in both cell lines, suggesting that the assembly of a complex made up of AR and Src drives the androgen-induced motility and invasiveness. Co-immunoprecipitation experiments in androgen-treated MDA-MB231 and MDA-MB453 cells show that the AR/Src complex recruits p85α, the regulatory subunit of PI3-K. In such a way, the basic machinery leading to migration and invasiveness is turned-on. The S1 peptide inhibits motility and invasiveness of TNBC cells and disrupts the AR/Src/p85α complex assembly in MDA-MB231 cells. This study shows that the rapid androgen activation of Src/PI3-K signaling drives migration and invasiveness of TNBC cells and suggests that the S1 peptide is a promising therapeutic option for these cancers.

## Introduction

Breast cancer (BC) is the most common cancer amongst women worldwide and despite considerable diagnostic and therapeutic efforts still represents the fifth leading cause of cancer-related mortality overall. Currently, immunohistochemistry and gene expression analysis are used to investigate the presence of ER, PR and HER2, which represent key targets in most of therapeutic protocols^1^.

Although significant progresses have been made for BC treatment, such as the development of anti-estrogen and anti-HER2 therapies, the disease frequently acquires drug-resistance, relapses and metastasizes^2,3^. To make even more complex the BC molecular landscape, it has been identified a specific BC subtype, not expressing ER or PR and characterized by the absence of HER2 overexpression/amplification. These cancers are commonly defined triple negative breast cancers (TNBCs) and account for approximately 10–20% of all BCs^4^. TNBCs early relapse and spread, thus, they are frequently associated with worse prognosis and a 5-year survival in 20–30% of patients. Unfortunately, there are not specific treatment guidelines for TNBCs and systemic chemotherapy still represents the only therapeutic option in both the early and advanced-stages of the disease. Therefore, new therapeutic strategies are needed for TNBCs^4^.

High-throughput approaches have identified several therapeutic targets in TNBC, such as the effectors of PI3-K- or Ras-dependent pathways. Targeted agents under clinical investigation include, indeed, PI3-K pathway or MEK inhibitors or their combination. Further, a TNBC subtype is characterized by the expression of luminal androgen receptor (LAR) in the presence of a luminal-like expression signature. This finding raises the question as to whether these cancers might be treated with agents that target AR, such as anti-androgens. Despite the accumulating studies, however, the role of AR in TNBC still remains debated^5–7^.

AR is a ligand-activated transcription factor that exerts its effects through genomic^8^ or non-genomic^9,10^ actions. The non-genomic model proposes that the androgen/AR axis drives rapid changes in membrane flexibility, [Ca2+] efflux and activation of second messenger pathways. Depending on the cellular milieu and ligand stimulation, activation of non-genomic pathways triggers different biological responses, such as proliferation, cell cycle progression, survival, invasiveness, differentiation and neuritogenesis^11^. Under different experimental conditions and in various cell types, including BC cells, the AR non-genomic action also mediates intersection of the receptor with growth factors receptors, such as the epidermal growth factor receptor (EGF-R; ^12,13^), the insulin growth factor receptor type I (IGF-R I; ^14^), the nerve growth factor receptor, TrkA^15,16^.

In this report, we have investigated the effect of androgens on motility and invasiveness of TNBC-derived cells. MDA-MB231 and MDA-MB453 cells that represent the mesenchyme and the LAR subtype of TNBC, respectively^17,18^ have been used. As these cells express AR, we have investigated whether androgens activate rapid signaling pathways involved in cell invasiveness. We found that the non-aromatizable androgen, R1881, triggers the AR-mediated migration and invasiveness of these cells. The anti-androgen bicalutamide and siRNA AR experiments indicate that the receptor mediates the observed effects. Notably, the small peptide S1, which perturbs the AR/Src complex assembly, impairs the androgen-dependent cell motility and invasiveness.

Our results identify a previously uncharacterized role for the hormone-regulated AR/Src complex assembly and the consequent invasiveness of TNBC cells. They also indicate that the AR/Src complex and its dependent pathway might represent a good network to target using the new S1 peptide in preclinical and clinical models of TNBC.

## Results

### Properties of AR in MDA-MB231 and MDA-MB453 cells

**Figure 1:**
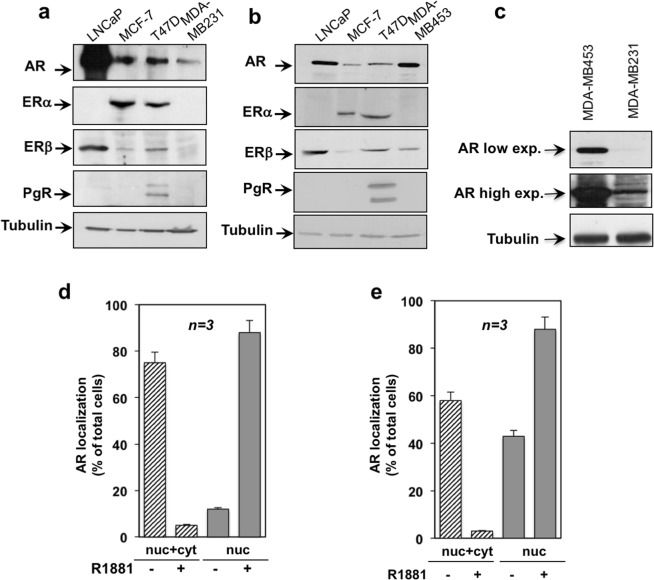
MDA-MB231 and MDA-MB453 cells express AR. In (**a–c**), lysates were prepared from the indicated cell lines and electrophoretically separated proteins were analyzed by Western blot, using the antibodies against the indicated proteins. In (**c)**, the amount of AR expressed in MDA-MB231 or MDA-MB453 cell lines was revealed by Western blot, using the anti AR antibody. A low and a high exposure blots for AR are shown. Quiescent MDA-MB231 (**d**) and MDA-MB453 (**e**) cells on coverslips were unchallenged or challenged with 10 nM R1881 for 30 min, then stained for AR and analyzed by IF, as described in Methods. Cells showing nucleo-cytoplasm (nuc + cyt) or exclusively nuclear (nuc) AR fluorescence were scored and quantified. Results from three different experiments were collected and expressed as % of the total cells. Means and SEMs are shown. *n* represents the number of experiments. For both cell lines, the difference in nuc + cyt or nuclear AR between the untreated cells and those challenged with 10 nM R1881 was significant (*p* < 0.05).

The expression of sex steroid receptors in human breast cancer MDA-MB231 and MDA-MB453 cells was analyzed by Western blot technique. It was performed using the 441 mouse monoclonal anti-AR antibody, directed to an epitope endowed within the 299–315 amino acid sequence of AR. Consistent with previous findings^18,19^, the antibody revealed an intense 110 KDa band in lysates from both cell lines. Of note, the amount of AR expressed in MDA-MB453 was higher than that observed in MDA-MB231 (Fig. 1, panel c) cells and similar to that detected in prostate cancer-derived LNCaP cells (Fig. 1, panel b). Neither estrogen receptor (ERα or ERβ) nor progesterone receptor (PgR A or PgR B) were detected in MDA-MB231 (panel a). A low level of ERβ was expressed in MDA-MB453 cell lysates (panel b), in the absence of ERα or PgR (PgR A or PgR B). As positive control, lysates from MCF-7, LNCaP and T47D cells were analyzed (Fig. 1a,b).

AR subcellular localization was then investigated by immunofluorescence (IF) staining with anti-AR antibody. The quantitative score shows that under basal conditions, the majority (about 80%) of MDA-MB231 exhibited nucleo-cytoplasmic staining of AR (Fig. 1d), with few cells exhibiting a prevalently nuclear AR localization. Under the same experimental conditions, 58% of MDA-MB453 cells showed nucleo-cytoplasmic localization of AR, with a significant number (42%) of cells exhibiting AR nuclear score. The finding that AR is predominantly localized in nucleo-cytoplasm of untreated MDA-MB231 might be due to the lower levels of AR expressed in these cells, as compared with MDA-MB453 cells. We previously showed that AR subcellular localization strictly depends on the receptor levels and ligand concentration^20^. In both cell lines, however, androgen stimulation induced within 30 min a robust AR nuclear translocation (panels d and e in Fig. 1). Images representative from one experiment in d and e were captured and shown in Fig. 1S. IF analysis in Fig. 1S also indicates that the antibody we used to detect AR is specific, since no fluorescence was detected in cells stained only with the fluorescein-conjugated secondary antibody, as a control (Fig. 1S).

**Figure 2:**
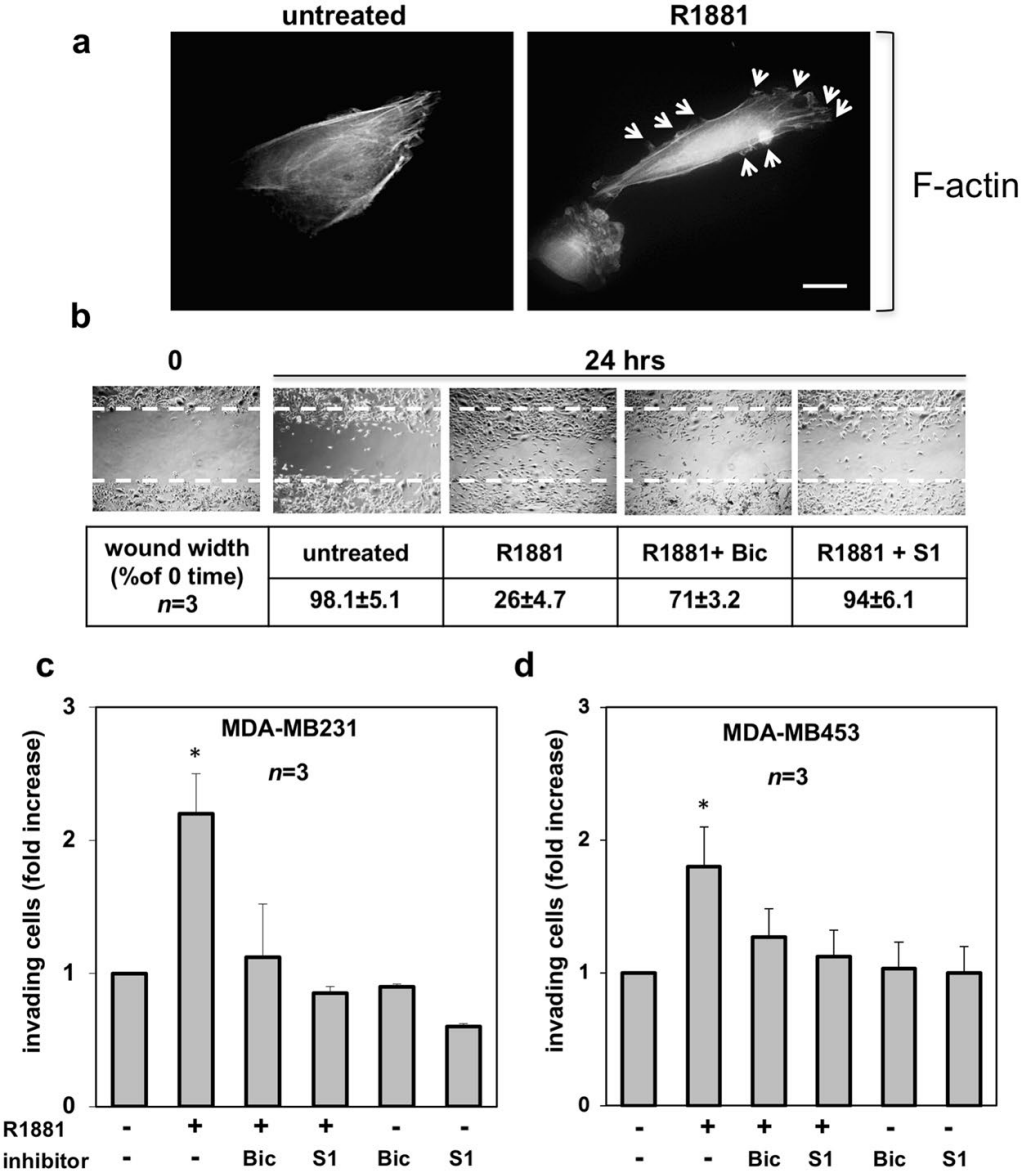
Androgen-triggered cytoskeleton changes, migration and invasiveness in MDA-MB231 and MDA-MB453 cells. In (**a**), quiescent MDA-MB231 cells were plated on coverslips and then left un-stimulated or stimulated for 20 min with 10 nM R1881. Thereafter, the cells were fixed, stained for F-actin using Texas-red conjugated phalloidin and analyzed by IF. Images representative of three independent experiments were captured and shown. The arrows indicate protrusions and ruffles in androgen-treated cells. Bar, 5μm. In (**b**), quiescent MDA-MB231 cells at sub-confluence were wounded and then left unstimulated or stimulated with R1881 (at 10 nM) for the indicated times. Cytosine arabinoside was included at 50 μM (final concentration) in the cell medium to avoid cell proliferation. About 14% of MDA-MB231 cells incorporated BrdU under quiescence condition. Challenging with 10 nM R1881 increased to 34% the number of cells incorporating BrdU, while simultaneous treatment with cytosine arabinoside reverted to 16% such number. When indicated, bicalutamide (Bic; at 1μM final concentration) and the S1 peptide (at 10 nM final concentration) were added 30 min before hormone stimulation. Contrast-phase images are representative of 3 different experiments, each in duplicate. Inset in (**b**), the wound area was measured using Image J Software and data are presented as % in wound width over the control, untreated cells (0 time). Data from 3 different experiments were collected and analyzed. Means and SEMs are shown. *n* represents the number of experiments. Quiescent MDA-MB231 (**c**) and MDA-MB453 (**d**) cells were used for invasion assay in Boyden’s chambers pre-coated with growth factor-reduced and phenol red-free Matrigel. The indicated compounds were added to the upper and the lower chambers. R1881 was used at 10 nM and bicalutamide (Bic) at 1μM. The S1 peptide was added (at 10 nM) 30 min before the hormonal stimulation. Here again, cytosine arabinoside (at 50 μM) was included in cell medium. About 12% of MDA-MB453 cells incorporated BrdU under quiescent, basal conditions. Challenging with 10 nM R1881 increased to 29% the number of MDA-MB453 cells incorporating BrdU, while simultaneous treatment with cytosine arabinoside reverted to 13% such number. After 24 h (for MDA-MB231 cells) or 36 h (for MDA-MB453 cells), invading cells from at least 30 fields /each membrane were counted as reported in Methods, using a DMBL (Leica) fluorescent microscope, equipped with HCPL Fluotar 20x objective. Results from three different experiments were collected and expressed as fold increase. Means and SEMs are shown. *n* represents the number of experiments. *p < 0,05 for the indicated experimental points *versus* the corresponding untreated control.

We also analyzed the AR subcellular localization by Western blot of cytoplasmic and nuclear enriched fractions from MDA-MB231 cells (panel a in Fig. 2S). The fractions were also probed for α-tubulin and histone H3, as cytoplasmic and nuclear marker, respectively. The upper panel in a (Fig. 2S) shows that androgen treatment increased over the time the bulk of AR levels in nuclear enriched fractions of MDA-MB231 cells. Cytoplasmic AR levels are undetectable both before and after hormone treatment. Again, this could be explained by the low level of AR in these cells, too diluted to be detectable by Western blot in cytoplasm.

We next evaluated the androgen-induced gene transcription. To this end, we used androgen-response element (ARE) reporter gene assay. After treating the cells with 10 nM R1881 or ethanol as a control, luciferase activity was analyzed by luciferase reporter gene assay. When treated with androgens, luciferase activity increased by 2-fold (*p* < 0.05) in MDA-MB231 cells (panel b in Fig. 2S) and by 2,2 fold (p < 0,05) in MDA-MB453 cells (panel c in Fig. 2S) transfected with the control pSG5 plasmid. A more robust (about 10,6 -fold) increase in luciferase activity was observed in MDA-MB231 cells transiently transfected with the wild type hAR-encoding plasmid (panel c in Fig. 2S). A slight (3-fold) but significant (p < 0,05) increase in luciferase activity was detected in MDA-MB453 cells transiently transfected with the wild type hAR-encoding plasmid (panel c in Fig. 2S). Differences in the expression and activity of co-regulators, fluctuation in ligand concentration and presence of endogenous inhibitors might contribute to the cell- and target gene-selective regulation by steroid receptors^21^. The corresponding lysates from cells transfected with the empty pSG5 plasmid or pSG5 encoding for the wild-type hAR were analyzed by Western blot for AR expression (inset in b and c; Fig. 2S).

This set of experiments shows that MDA-MB231 and MDA-MB453 cells harbor a classic AR, migrating at 110 KDa, which undergoes nuclear translocation and mediates gene transcription activation upon androgen challenging of cells.

### Androgens trigger cytoskeleton changes, migration and invasiveness in TNBC cells

AR mediates rapid responses leading to motility and invasiveness of target cells^20,22^. Since changes in the actin cytoskeleton are hallmarks of migrating cells^23,24^, we firstly analyzed the actin remodeling upon androgen treatment of MDA-MB231 cells. Ten nM R1881 treatment induced within 20 minutes ruffles and protrusions (marked by arrows) in quiescent MDA-MB231 cells (Fig. 2a). These changes were dynamically shut off within 1 h (not shown).

To determine whether these changes affect cell migration, quiescent MDA-MB231 cells were wounded and allowed to migrate in the absence or presence of the indicated compounds. Representative images from one experiment were captured by contrast-phase microscopy (Fig. 2b). Different experiments were done and the wound width was analyzed. Data are presented in panel b (inset). Collectively, the results in b show that a significant number of cells migrated in the wound area upon 10 nM R1881 treatment, as compared with untreated cells. Bicalutamide (Bic) inhibited the androgen-induced effect, indicating that the classical AR is implied in this response. By extending the bicalutamide treatment time to 48–72 hours, we still observed the inhibitory effect on cell migration (not shown). The peptide S1 that inhibits the association of AR with SH3-Src domain^25^ also impaired cell motility (b and inset).

We did not use wound scratch assay in MDA-MB453 cells, since these cells are commonly considered semi-adherent^26,27^. Therefore, we analyzed the androgen effect in TNBC invasiveness, using a Matrigel transmigration assay. Androgens increased by about 2,2 and 1,8 fold the number of MDA-MB231 (panel c in Fig. 2) and MDA-MB453 (panel d in Fig. 2) invading cells, respectively. Bicalutamide and S1 peptide significantly (p < 0,05) inhibited this effect, while they scantly affected the number of invading cells in the absence of hormone.

Altogether, findings in Fig. 2 indicate that classic AR mediates the hormonal effect on cell migration and show that S1 peptide impairs the androgen-triggered invasiveness in TNBC cells.

### AR mediates the androgen-triggered invasiveness of TNBC cells

**Figure 3:**
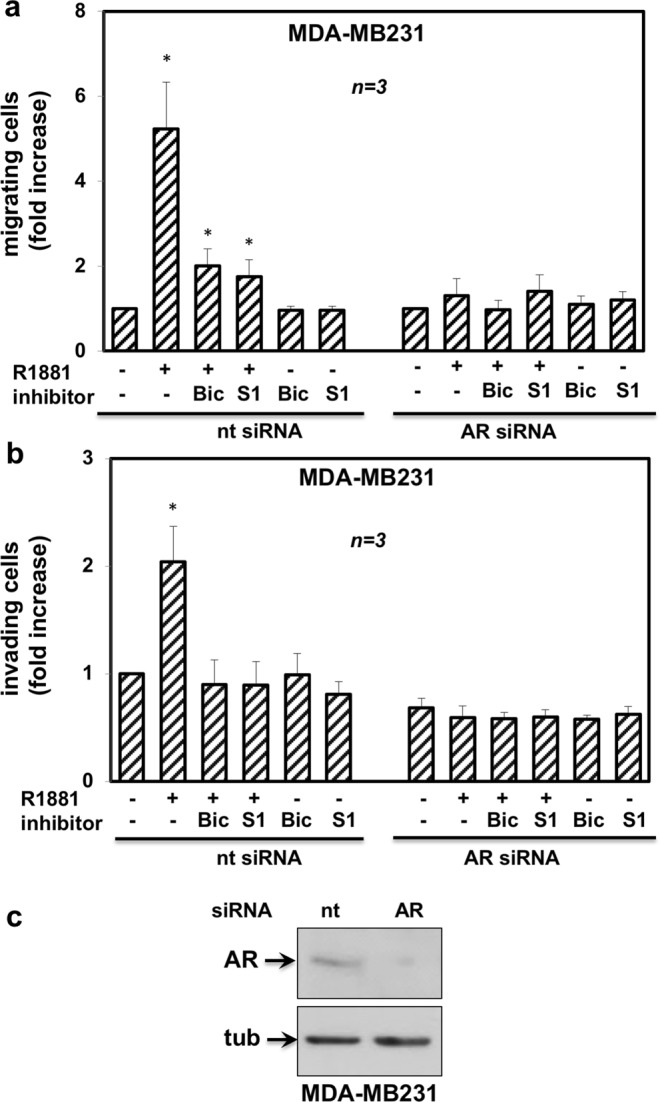
AR is required for androgen-induced motility and invasiveness of MDA-MB231 cells. MDA-MB231 cells were transfected with AR siRNA or non-targeting siRNA, using Lipofectamine^TM^ 2000. siRNA Alexa Fluor 488 was included to identify transfected cells. After transfection, cells were made quiescent for 24 h, then used for migration (**a**) or invasion (**b**) assays. In (**a**), migration assay was performed in Boyden’s chambers pre-coated with collagen. In (**b**), invasion assay was performed in Boyden’s chambers pre-coated with growth factor-reduced and phenol red-free Matrigel. In both assays, the indicated compounds (R1881 at 10 nM, bicalutamide at 1μM, S1 peptide at 10 nM and cytosine arabinoside at 50 μM) were added to the upper and the lower chambers. In (**a**), transfected cells were allowed to migrate for 18 h and then fixed in 4% paraformaldehyde for 20 min. In (**b**), transfected cells were allowed to invade for 24 h. Alexa Fluor 488-migrating (**a**) or invading (**b**) cells, then scored and counted using a DMBL (Leica) fluorescent microscope equipped with HCPL Fluotar 20x objective in 30 random microscopic fields. In (**a**,**b**), results were collected and expressed as fold increase. Means and SEMs are shown. ***n*** represents the number of experiments. *p < 0,05 for the indicated experimental points *versus* the corresponding untreated control. In (**c**), lysates from MDA-MB231 cells transfected with AR siRNA or non-targeting siRNA were prepared. Proteins were separated by SDS-PAGE and then analyzed by Western blot, using the antibodies against the indicated proteins.

**Figure 4:**
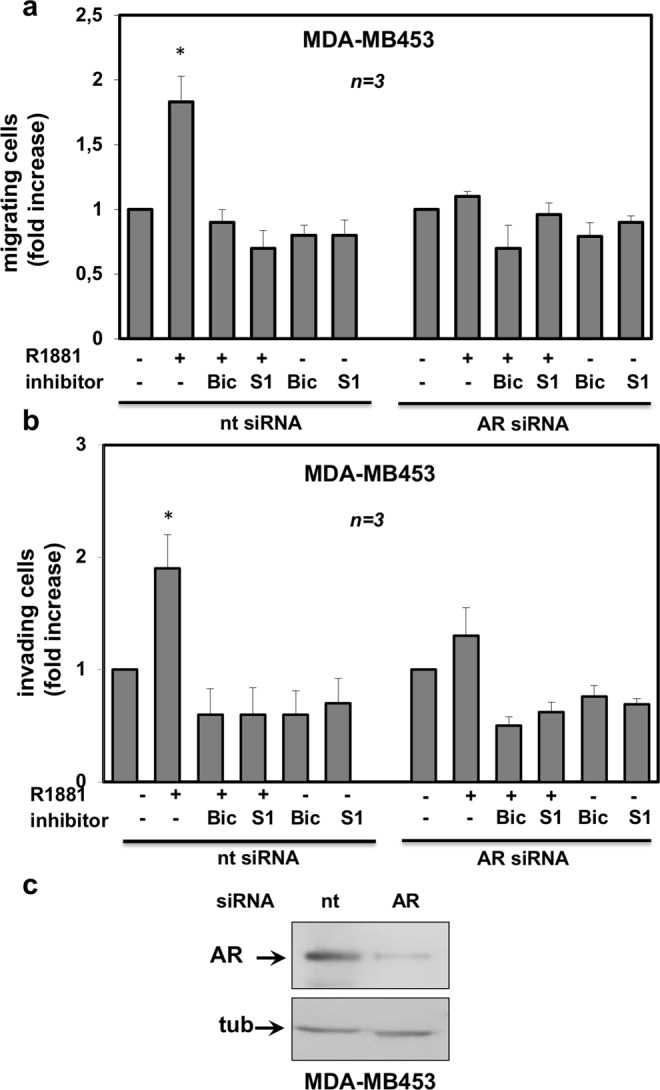
AR is required for androgen-induced motility and invasiveness of MDA-MB453 cells. MDA-MB453 cells were transfected with AR siRNA or non-targeting siRNA, using Lipofectamine^TM^ 2000. siRNA Alexa Fluor 488 was included to identify transfected cells. After transfection, the cells were made quiescent for 24 h and then used for migration (**a**) or invasion (**b**) assays. In (**a**), migration assay was done in Boyden’s chambers pre-coated with collagen. In (**b**), invasion assay was done in Boyden’s chambers pre-coated with growth factor-reduced and phenol red-free Matrigel. In both assays, the indicated compounds (R1881 at 10 nM, bicalutamide at 1μM, S1 peptide at 10 nM and cytosine arabinoside at 50 μM) were added to the upper and the lower chambers. In (**a**), transfected cells were allowed to migrate for 24 h and then fixed in 4% paraformaldehyde for 20 min. In (**b**), transfected cells were allowed to invade for 36 h. Alexa Fluor 488-migrating (**a**) or invading (**b**) cells were finally scored and counted using a DMBL (Leica) fluorescent microscope equipped with HCPL Fluotar 20x objective in 30 random microscopic fields. In (**a**,**b**), results were collected and expressed as fold increase. Means and SEMs are shown. *n* represents the number of experiments. *p < 0,05 for the indicated experimental points *versus* the corresponding untreated control. In (**c**), lysates from MDA-MB453 cells transfected with AR siRNA or non-targeting siRNA were prepared. Proteins were separated by SDS-PAGE and then analyzed by Western blot, using the antibodies against the indicated proteins.

MDA-MB231 and MDA-MB453 cells express significant levels of glucocorticoid receptor (GR) and R1881 also binds GR^28–30^. GR might, hence, substitute for AR in promoting cell invasiveness upon androgen stimulation. To address this issue, we inhibited the AR-mediated activity by knockdown experiments. Silencing of AR by siRNA drastically and significantly (p < 0,05) lowered the number of MDA-MB231 migrating (Fig. 3a) or invading (Fig. 3b) cells upon androgen stimulation. In contrast, transfection of cells with non-targeting, control siRNA did not abolish the androgen stimulatory effects. Expectedly, bicalutamide and S1 peptide both abolished the androgen-triggered motility (panel a) or invasiveness (panel b) of MDA-MB231 cells transfected with ctrl siRNA. The Western blot analysis in Fig. 3c shows that AR was actually silenced in AR siRNA transfected cells, as compared with control cells, transfected with non-targeting siRNA. At last, we inhibited the AR-mediated activity by knockdown experiments in MDA-MB453. Results superimposable to those obtained in MDA-MB231 were observed in both migration (Fig. 4a) and invasiveness (Fig. 4b) assays. Here again, the Western blot analysis in Fig. 4c shows that AR was abolished in AR siRNA MDA-MB453 transfected cells, as compared with control cells, transfected with non-targeting siRNA.

Findings in Figs 3 and 4 definitely show that AR, but nor GR, mediates the hormone-induced migration and invasiveness of TNBC cells. Results obtained using both bicalutamide and S1 peptide further reinforce this issue.

### Androgen-induced invasiveness in MDA-MB231 cells: role of the AR/Src/PI3-K/FAK complex assembly and its downstream pathway activation

**Figure 5:**
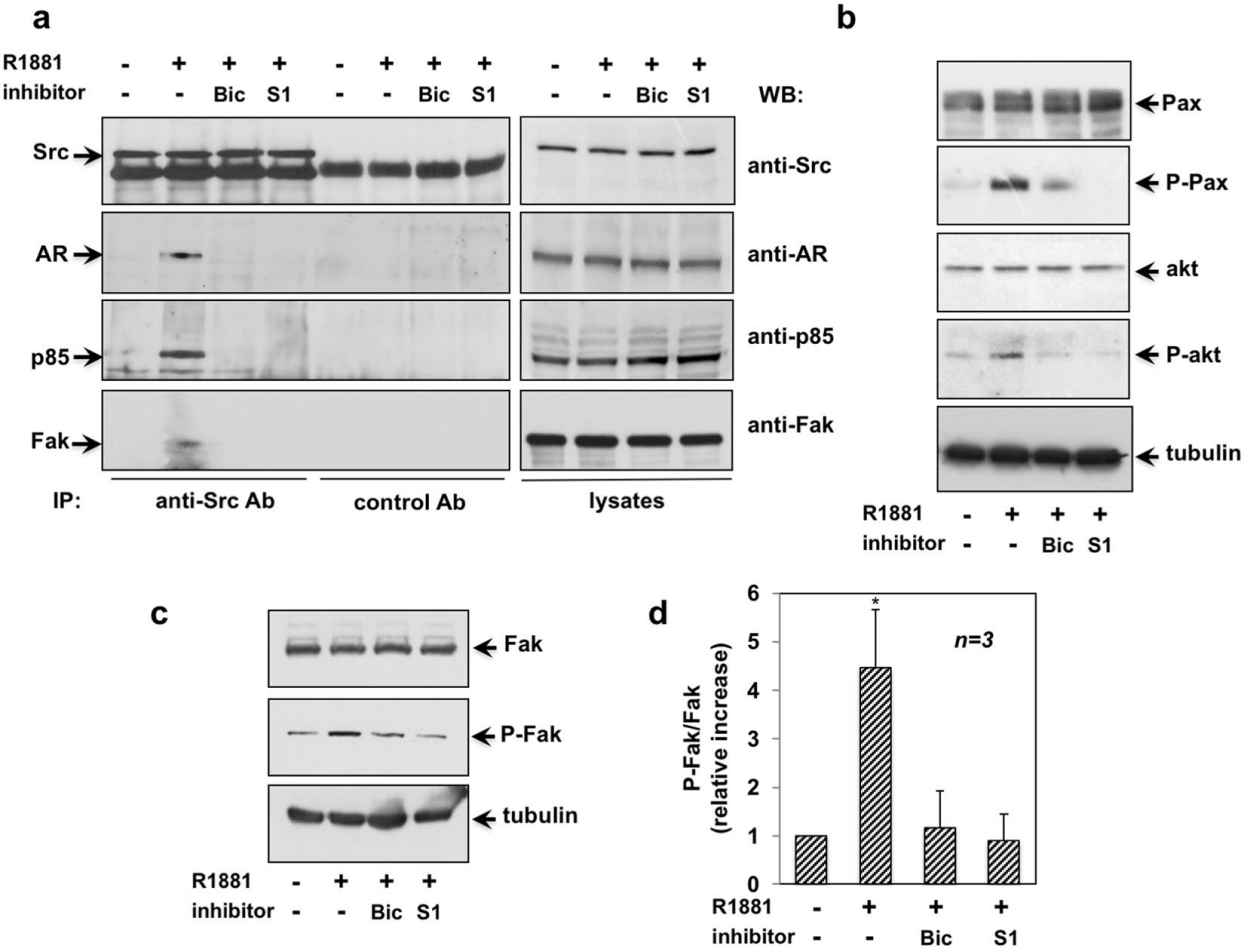
Androgen-stimulated invasiveness of MDA-MB231 cells: role of the AR/Src/PI3-K/FAK complex. Quiescent MDA-MB231 cells were used. In (**a**–**d**), the cells were stimulated for 5 min with 10 nM R1881, in the absence or presence of the indicated compounds. Bicalutamide (Bic; at 1μM) and the S1 peptide (at 10 nM) were added 30 min before the hormone stimulation. In a (left panels), cell lysates were immune-precipitated using anti Src antibody (anti Src Ab) or control IgG (control Ab). Proteins in immune-complexes were analyzed by Western blot, using the antibodies against the indicated proteins. Right panels in a and panel b, lysates were prepared and electrophoretically separated proteins were analyzed by Western blot, using the antibodies against the indicated proteins. P-Pax stands for Tyr 118-P-Paxillin; and p-Akt for P-Ser 473 Akt. The filter in (**b**) was stripped and re-probed using anti tubulin antibody, as loading control. In (**c**), lysates were prepared and electrophoretically separated proteins were analyzed by Western blot, using the antibodies against total Fak or P-Tyr397 Fak. The filter was stripped and re-probed using anti tubulin antibody, as a loading control. In (**d**), analysis of Fak activation was done in three different experiments. For each experiment, the ratio P-Fak/Fak was evaluated using the NIH Image J program. Results were expressed as relative increase. Means and SEMs are shown. *n* represents the number of experiments. *p < 0,05 for the indicated experimental point *versus* the corresponding untreated control.

Since the S1 peptide displaces the AR/Src interaction in androgen-treated cells^25^, we hypothesized a role for this complex in the observed effects. Therefore, we immune-precipitated Src tyrosine kinase from lysates of MDA-MB231 cells challenged with the compounds indicated in Fig. 5a. Co-immune-precipitated proteins were then analyzed by Western blot. Irrespective of cell treatments, the same amount of Src was collected in immune-precipitated proteins (left panel in Fig. 5a). Androgens induced the recruitment of AR to Src (left panel in Fig. 5a). We also detected in the complex the p85α regulatory subunit of PI3-K (left panel in Fig. 5a) and the Src substrate, FAK (left panel in Fig. 5a). Bicalutamide and S1 peptide both disrupted the assembly of this multi-molecular complex. Again, the IP approach is specific, since no proteins were detected in lysates precipitated with control antibodies. Lastly, the corresponding cell lysates were probed with the indicated antibodies to verify that similar amount of proteins were immune-precipitated (right panels in a).

FAK and the focal contact-associated protein paxillin are involved in cell adhesion and motility, and both proteins can be phosphorylated on tyrosine residues^31,32^. Figure 5b shows that androgen rapidly (within 5 min) triggered paxillin Tyr 118-phosphorylation in MDA-MB231 cell lysates (upper panel). The PI3K/Akt pathway is actively involved in cancer cell motility and metastasis. In agreement with these findings, we observed androgen-triggered Ser-473 phosphorylation of Akt (lower panel). Here again, the androgen effect on activation/phosphorylation of paxillin and Akt was significantly blocked by the S1 peptide. Albeit at lesser extent, bicalutamide also inhibited the androgen effect on the phosphorylation of the same effectors. At last, we detected a significant increase of FAK Tyr 397-phosphorylation in androgen-treated cells. S1 peptide and bicalutamide both inhibited such activation (Fig. 5c). Densitometry analysis of P-FAK was done in different experiments and collected data are presented in Fig. 5d.

Thus, androgens induce migration and invasiveness of MDA-MB231 cells through the assembly of AR/Src/PI3-K/FAK complex. Once assembled, such a complex leads to phosphorylation of FAK, Akt and paxillin The S1 peptide that displaces the upstream AR/Src/PI3-K/FAK complex also inhibits phosphorylation of FAK, Akt and paxillin. It is through these effects that S1 likely impairs cell migration and invasiveness.

### AR is required for the androgen-induced Src/PI3-K/FAK complex assembly and activation of the downstream pathways in MDA-MB231 cells

**Figure 6:**
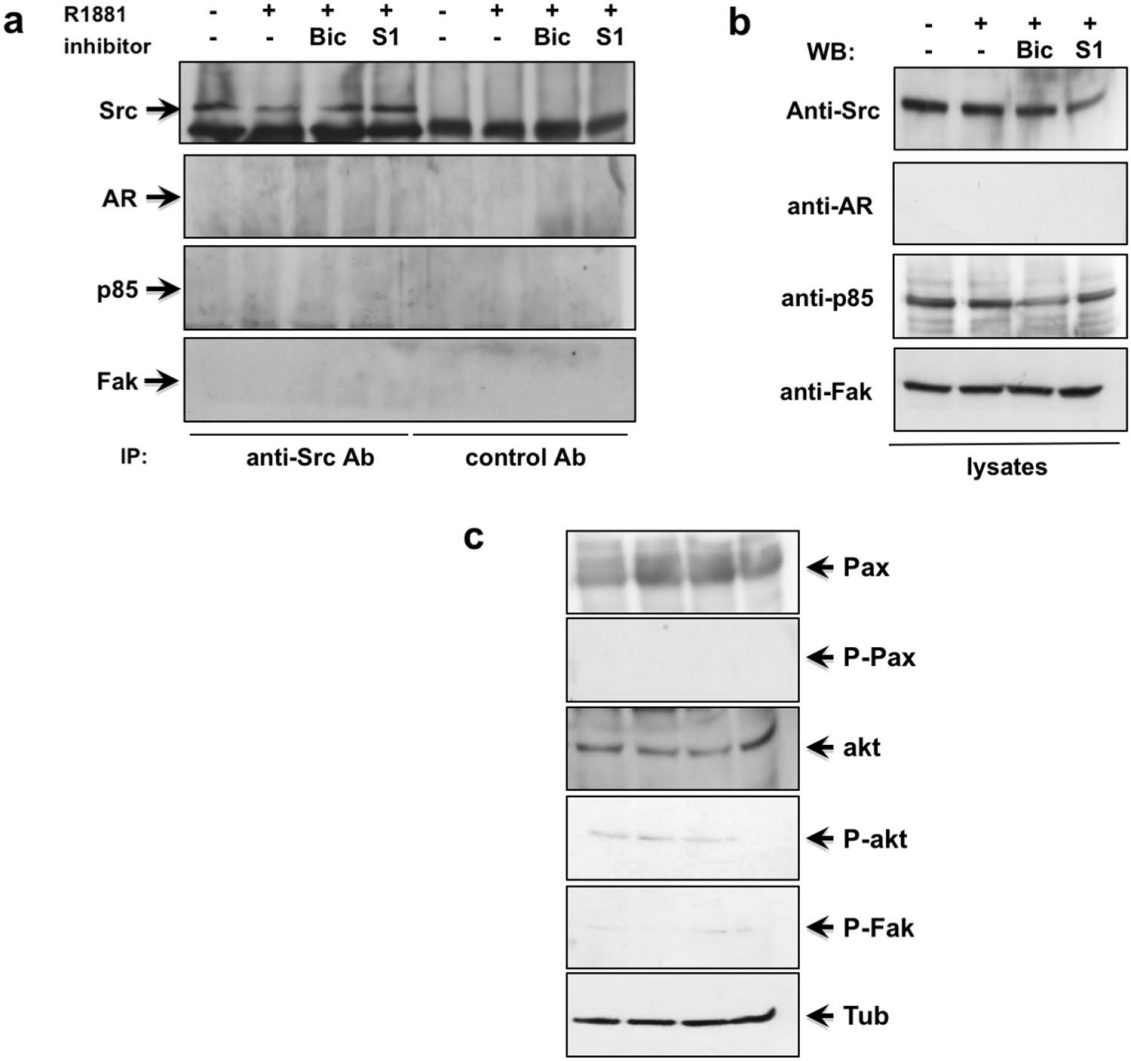
AR is required for androgen-induced AR/Src/PI3-K/Fak complex in MDA-MB231 cells. MDA-MB231 cells were transfected with AR siRNA (**a**–**c**), using Lipofectamine^TM^ 2000. After transfection, the cells were made quiescent for 24 h and then left unchallenged or challenged for 5 min with 10 nM R1881, in the absence or presence of the indicated compounds. Bicalutamide (Bic; at 1μM) and the S1 peptide (at 10 nM) were added 30 min before the hormone stimulation. Cell lysates were prepared. In (**a**), lysates were immune-precipitated using the anti Src antibody (Anti Src Ab) or control IgG (control Ab). Proteins in immune-complexes were analyzed by Western blot, using the antibodies against the indicated proteins. In (**b**,**c**), lysates proteins were analyzed by Western blot, using the antibodies against the indicated proteins. P-Pax stands for Tyr 118-P-Paxillin, P-akt for P-Ser 473 Akt and P-Fak stands for P-Tyr397 Fak. Filters were stripped and re-probed using anti tubulin antibody, as loading control.

Here again, we inhibited the AR-mediated activity by knockdown experiments in MDA-MB231 (Fig. 6a–c). Control cells were transfected with non-targeting siRNA (Fig. 3S, a–c). Cells were left unchallenged or challenged with 10 nM R1881, in the absence or presence of bicalutamide or S1 peptide. Src tyrosine kinase was then immune-precipitated from cell lysates and co-immune-precipitated proteins were finally analyzed by Western blot. In cells transfected with siRNA AR (Fig. 6a) or non-targeting siRNA (Fig. 3S, a), the same amount of Src tyrosine kinase was collected by IP. Neither AR, nor p85α nor FAK were recruited by Src in cells transfected with siRNA AR (Fig. 6a), regardless of hormone stimulation and presence of inhibitors. Notably, the androgen-induced complex assembly was restored only in androgen-stimulated MDA-MB231 cells transfected with non-targeting siRNA, as control (Fig. 3S, a).

As readout for androgen-triggered Src/AR/PI-3K/FAK complex assembly, we looked at the androgen activation of the downstream signaling effectors. In MDA-MB231 cells, AR silencing reverted the stimulatory effect of androgens on paxillin, Akt and Fak activation (Fig. 6c). Expectedly, a robust activation of these effectors was observed in cells transfected with non-targeting siRNA and challenged with androgens, as control (Fig. 3S, c). Regardless of AR silencing, similar amounts of signaling effectors were detected in total cell lysates (Fig. 6b and Fig. 3S, b). AR knockdown by siRNA completely abolished the AR expression in MDA-MB231 cells (Fig. 6b). Irrespective of experimental condition, similar AR levels were revealed in cells transfected with non-targeting siRNA (Fig. 3S, b).

Findings in Fig. 6 definitely show that AR mediates the androgen-induced Src/PI3-K/FAK complex assembly and activation of its dependent pathway in MDA-MB231 cells.

### Androgen rapidly induces AR/Src/PI3-K complex assembly and FAK activation in MDA-MB453 cells

**Figure 7:**
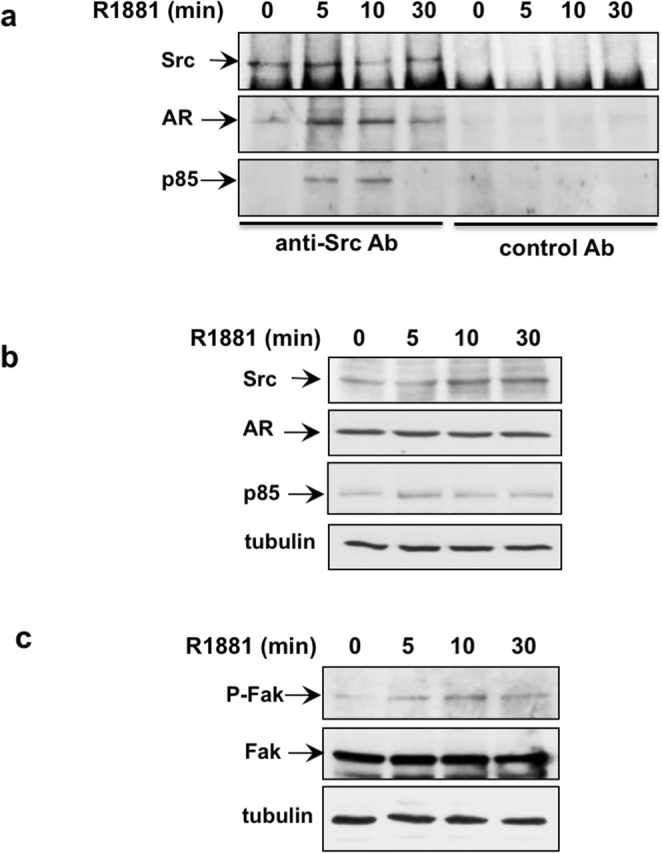
Androgens induce AR/Src/PI3-K complex assembly and FAK activation in MDA-MB453 cells. Quiescent MDA-MB453 cells were used. In (**a**–**c**), cells were stimulated for the indicated times with 10 nM R1881. In (**a**), lysate proteins (5 mg/ml) were immune-precipitated using the anti Src antibody (Src) or control IgG (IgG mouse). Proteins in immune-complexes were analyzed by Western blot, using the antibodies against the indicated proteins. In (**b**,**c**), lysate proteins were analyzed by Western blot, using the antibodies against the indicated proteins. P-Fak stands for P-Tyr397 Fak. The blot in (**c**) is representative of two different experiments. The filters were stripped and re-probed using anti tubulin antibody, as loading control.

Luminal BC-derived MDA-MB453 cells express very low, almost undetectable levels of Src tyrosine kinase^33^. Therefore, we used protein-enriched MDA-MB453 cell lysates to detect the AR/Src/PI3K complex in these cells. Quiescent cells were left untreated or treated for the indicated times with androgens and lysate proteins (5 mg/ml protein concentration) were immune-precipitated with anti Src or mouse IgG control antibody (Fig. 7a). Albeit similar amounts of Src tyrosine kinase were collected in immune-precipitated proteins, 5 min androgen treatment induced recruitment of AR and the p85 alpha to Src. Such effect persisted until 10 min to decline upon 30 min hormone treatment (Fig. 7a). Similar amounts of Src, or AR or p85 alpha were detected in loaded lysate proteins (Fig. 7b). As readout for androgen-triggered Src/AR/PI3K complex, we detected FAK activation by androgens in the same cells (Fig. 7c). We did not assay Akt activation, since MDA-MB453 cells harbor genetic lesions that activate PI3K/Akt signaling^34^.

In conclusion, androgens promote the assembly of AR/Src/PI3K complex in MDA MB453 cells. It is through disruption of this complex that S1 peptide likely impairs migration and invasiveness in these cells (see Figs 2 and 4).

## Discussion

Up to 80% of human BC are positive for AR and 25% of BC metastases express AR, whereas ER and PR levels are almost undetectable^35,36^. This latter finding suggests a role for AR in BC invasiveness and spreading. Evidence so far collected suggests that AR fosters BC proliferation when ER is expressed, while it is associated with a worse outcome and a higher risk of recurrences in TNBC^37^. Despite the accumulating findings, the role of androgen/AR axis in BC still remains controversial.

In this paper, we have investigated the androgen non-genomic mechanism leading to invasiveness of MDA-MB231 and MDA-MB453 cells. These cells harbor a classic AR, as shown by its ability to translocate into the nuclear compartment and activate gene transcription. The ligand activated-receptor also mediates cell motility and invasiveness, as assessed by both wound scratch and Matrigel trans-migration assays. These findings are reminiscent of the androgen effect on cytoskeleton rearrangements previously reported in BC-derived T47D cells^38^. Bicalutamide inhibits cell motility and invasiveness, indicating the involvement of classic AR in the observed responses. siRNA experiments definitely confirm this issue and exclude the possibility that R1881 acts through GR in our experimental setting. Data obtained using the small S1 peptide, further support this issue, since the peptide, which mimics a proline-rich stretch of AR sequence, specifically perturbs the interaction of AR with the SH3-Src domain^25,39^. The peptide almost completely impairs motility and invasiveness of cells, implicating that the androgen-triggered AR/Src complex assembly controls these responses in TNBC cells. Co-immunoprecipitation experiments confirm that androgen treatment of quiescent MDA-MB231 and MDA-MB453 cells rapidly induces the assembly of AR/Src complex. S1 peptide disrupts this complex in MDA MB231 and likely in MDA MB453 cells. In addition, siRNA experiments corroborate the requirement for AR in the complex assembly.

The bipartite AR/Src complex controls prostatic cancer cell proliferation *in vitro* or in nude mice xenografts^25,39^. Additional findings have shown that the tumor suppressor DOC2/DAB2 (differentially expressed in ovarian cancer 2/disabled 2′) counteracts the AR/Src complex assembly and attenuates its oncogenic properties^40^. The role of this complex in prostate transformation has been also investigated using an *in vivo* prostate regeneration system^41^. Altogether, these findings support a role for this complex in transformation of androgen-responsive tissues.

**Figure 8:**
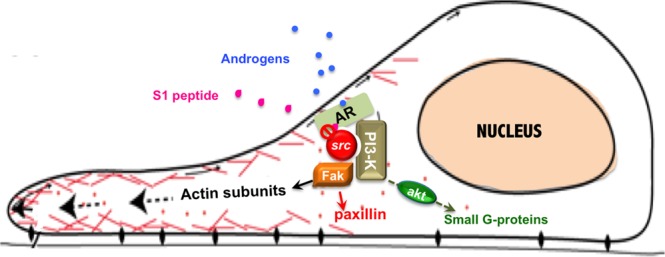
Model of androgen-induced invasiveness in TNBC cells. Androgen stimulation of TNBC cells rapidly triggers the AR/Src complex assembly. Once assembled, this complex recruits PI3-K and FAK. PI3-K activation leads to Ser 473 phosphorylation of Akt, while FAK undergoes phosphorylation on Tyr 397. This latter event likely leads to phosphorylation of paxillin on Tyr-118. Once activated, all these effectors cooperate each other in modulating focal contact/focal adhesion turnover and invasiveness. The small peptide S1 perturbs the AR/Src complex assembly, thereby inhibiting the downstream pathways involved in cell invasiveness.

The androgen-induced AR/Src complex recruits p85α, the regulatory subunit of PI3-K, and FAK, a Src substrate involved in adhesion and migration. Bicalutamide and S1 peptide both perturb the assembly of this multi-molecular complex. Estrogen treatment of breast cancer MCF-7 cells or androgen challenging of mesenchyme cells induce the assembly of a similar complex, which impinges on cell cycle progression and ERα cytoplasmic localization^22,42–46^. Of note, other studies have indicated that a ternary complex made up of AR, p85α, and Src is required for androgen stimulation of the PI3K/Akt pathway, which mediates androgen-induced growth and survival of MCF7 and LNCaP cells^47^. We here report that this complex controls motility and invasiveness of TNBC cells, as a disruptor peptide inhibits these effects in two TNBC cell types, which are representative of mesenchyme (MDA-MB231 cells) and luminal (MDA-MB453 cells) phenotype. The AR/Src/PI3-K/FAK complex triggers FAK auto-phosphorylation and Tyr-118 paxillin phosphorylation, in one hand. On the other, it triggers Akt activation. Once activated, these effectors cooperate each other in controlling cell invasiveness (Fig. 8). Rapid activation of FAK and paxillin phosphorylation by sex-steroids has been observed in different cell types and linked to motility, invasiveness or survival of target cells^22,44,48,49^. Again, the role of Akt activation in cancer cell spreading is undeniable^50^. The findings here presented for the first time show that the AR/Src complex is upstream of androgen-activated non-genomic signaling that involves FAK, paxillin and Akt, thereby leading to migration and invasiveness of TNBC cells.

In addition to their novelty, our results have a potential application in BC therapeutic approach. LAR-BC, and hence MDA-MB453 cells, are often enriched of PI-K3CA and PTEN mutations. They exhibit, indeed, strong sensitivity to PI3-K inhibitors and androgen blockers^51,52^. As such, the utility of targeting both PI3-K and AR is being explored in a phase I/II study based on the combination of an androgen blocker with a PI3-K inhibitor in LAR-TNBC patients^5^. Again, the mesenchyme-like BC cells, and hence MDA-MB231 cells, are often enriched in the expression of genes encoding for the effectors involved in cell migration, such as FAK^52,53^. Since FAK activity depends on Src^53^, the Src inhibitor, dasatinib has been successfully used to block invasiveness properties of MDA-MB231 cells^54^. Despite the encouraging findings obtained in cultured cells, dasatinib has shown limited efficacy when used as a single-agent in TNBC patients^54^. Moreover, the anti-androgen therapies explored in LAR-TNBC patients have various side effects^55^ and frequently induce resistance^56^. At last, the finding that androgens rapidly trigger the AR/Src/PI3-K complex assembly in both LAR and mesenchyme subtype of TNBC cells suggests that assembly of this complex is related to AR expression, rather than to specific cellular properties exhibited by MDA-MB231 cells. Therefore, the AR/Src/PI3-K/FAK complex emerges as an appropriate target for BC therapy.

In conclusion, our study offers new insights for identification of biomarkers predictive of TNBC malignancy. Findings obtained by using the S1 peptide inhibitor of AR/Src complex may provide new hints for exploration of novel drugs in clinical application. Molecular therapies that target the upstream molecular partners of AR deserve further evaluation in preclinical and clinical models of TNBC.

## Methods

### Chemicals, reagents and constructs

R1881 and bicalutamide (Sigma-Aldrich) were used at 10 nM and 10 μM, respectively. The S1 peptide was designed, synthesized and purified as reported^25^. It was added (at 10 nM, final concentration) 30 minutes before hormonal stimulation. cDNA encoding the wild-type version of human AR (hAR) was cloned in pSG5, as previously described^57^. The 3416 construct containing four copies of wild-type *slp*-HRE2 (5′-TGGTCAgccAGTTCT-3′) was cloned in the *Nhe*I site in pTK-TATA-Luc, as reported^58^.

### Cell cultures

Human BC-derived cells (MDA-MB231, MDA-MB453, MCF-7 and T47D cells) and prostate cancer-derived LNCaP cells were from Cell Bank Interlab Cell Line Collection (ICLC- Genova - ITALY). Cells were maintained at 37 °C in humidified 5% CO_2_ atmosphere. MDA-MB231 cells were cultured in phenol-red DMEM containing 10% fetal bovine serum (FBS), penicillin (100 U/ml), streptomycin (100 U/ml) and glutamine (2 mM). Twenty-four hours before stimulation, growing MDA-MB231 cells at 70% confluence were made quiescent using phenol red-free DMEM medium containing 0,1% charcoal-stripped serum (CSS), penicillin (100 U/ml) and streptomycin (100 U/ml). In wound healing assay, the charcoal treatment of FBS was repeated three times, instead of the routine two times, to reduce contamination by steroids. MDA-MB453 cells were cultured in phenol-red DMEM/F12 containing 10% fetal bovine serum (FBS), penicillin (100 U/ml), streptomycin (100 U/ml), glutamine (2 mM) and insulin (10 μg/ml, Roche). Twenty-four hours before stimulation, growing cells at 70% confluence were made quiescent under conditions identical to those employed for MDA-MB231 cells. MCF-7 cells were cultured in DMEM supplemented with glutamine (2 mM), penicillin (100 U/ml), streptomycin (100 U/ml), insulin (6 ng/ml), hydrocortisone (3.75 ng/ml) and 5% FBS. T47D cells were cultured in RPMI-1640 supplemented with glutamine (2 mM), penicillin (100 U/ml), streptomycin (100 U/ml), insulin (6 ng/ml), hydrocortisone (3.75 ng/ml) and 10% FBS. LNCaP cells were cultured in RPMI-1640 supplemented with 10% FBS, glutamine (2 mM), penicillin (100 U/ml), streptomycin (100 U/ml), sodium pyruvate (1 mM) and non-essential amino acids (10 mM). Media and supplements were from Gibco. The cell lines employed were routinely monitored for *Mycoplasma* contamination and expression of steroid receptors. The hormone-dependence of MCF-7, T47D and LNCaP cell lines was routinely analyzed by BrdU incorporation assay, as previously reported^59,60^.

### Transfection, transactivation assay and siRNA experiments

Transactivation assay was done as reported^61^. Briefly, MDA-MB231 and MDA-MB453 cells were transfected with 4 μg of purified 3416-pTK-TATA-Luc, alone or with 1 μg of purified pSG5-hAR-expressing plasmid, using Lipofectamine (Lipo 2000-Gibco). After 6 hrs, transfected cells were made quiescent for 24 hrs and then left un-stimulated or stimulated with 10 nM R1881 for 18 hrs. Luciferase activity from lysates was measured using a luciferase assay system (Promega) and values were corrected using CH110-expressed β-galactosidase activity (GE Healthcare). Data were obtained from three independent experiments, each performed in triplicate. siRNA experiments were done as described^22^, using Lipofectamine^TM^ 2000 (Gibco). A pool of 4 target-specific 20–25 nt siRNAs (Santa Cruz) was used for AR siRNA. Non-targeting siRNAs, containing a scrambled sequence, was from Santa Cruz. When indicated, cells were co-transfected with siRNA Alexa Fluor 488 (Cell Signalling) to identify transfected cells. Six hours after transfection, the cells were made quiescent for 24 h and then used.

### Wound healing and invasion assays

For wound scratch assay, 2 × 10^5^ cells were seeded in a 24-well plate. The cells were starved for 24 hrs, wounded using 10 µl sterile pipette tips and then washed with PBS. The cells were left un-stimulated or stimulated for 24 h with 10 nM R1881 in the absence or presence of the indicated compounds. To avoid cell proliferation, cytosine arabinoside (Sigma) at 50 μM (final concentration) was included in the cell medium. Different fields were analyzed with DMIRB inverted microscope (Leica) equipped with N-Plan 10x objective (Leica). Contrast-phase images were captured using a DC200 camera (Leica) and acquired using Application Suite Software (Leica). Images are representative of at least three different experiments. Migration assay was done using 3 × 10^4^ cells in Boyden’s chambers with 8 μm polycarbonate membrane (Falcon) pre-coated with collagen. The indicated stimuli were added to the upper and the lower chambers. Cytosine arabinoside (at 50 μM) was included in the cell medium. After 18 h (MDA-MB231 cells) or 24 h (MDA-MB453 cells), non-migrating cells from the membrane upper surface were removed using a sterile cotton swab. Invasion assay was done using 5 × 10^4^ cells in Boyden’s chambers with 8 μm polycarbonate membrane (Falcon) pre-coated with growth factor reduced and phenol red-free Matrigel (Corning). The indicated stimuli were added to the upper and the lower chambers. Here again, cytosine arabinoside (at 50 μM) was included. After 24 h (for MDA-MB231 cells) or 36 h (for MDA-MB453 cells), non-invading cells from the membrane upper surface were removed using a sterile cotton swab. In both, migration and invasiveness assays, the membranes were fixed for 20 min in 4% paraformaldehyde, stained with Hoechst, removed with forceps from the companion plate and mounted. Migrating or invading cells from at least 30 fields /each membrane were counted using a DMBL (Leica) fluorescent microscope, equipped with HCPL Fluotar 20x objective. In siRNA AR studies, MDA-MB231 and MDA-MB453 cells were co-transfected with siRNA Alexa Fluor 488 to identify transfected cells. Migration and invasiveness assay was done as previously indicated. MDA-MB231 or MDA-MB453 cells transfected with siRNA Alexa Fluor 488 were fixed in 4% paraformaldehyde for 20 min. Alexa Fluor 488-migrating or -invading cells were finally scored and counted using a DMBL (Leica) fluorescent microscope equipped with HCPL Fluotar 20x objective in 30 random microscopic fields. Data are representative of at least three different experiments.

### Cytoskeleton analysis, AR staining and immunofluorescence

MDA-MB231 and MDA-MB453 cells were plated on gelatin-coated coverslips. For AR staining, cells on coverslips were fixed in 4% paraformaldehyde and permeabilized using diluted (0,2% in PBS) Triton-X100. The cells were then stained with diluted (1:25 in PBS) Alexa Fluor 488-conjugate rabbit monoclonal anti-AR antibody (Cell Signaling). When indicated, the secondary antibody Alexa Fluor 488 AffiniPure Goat Anti-Rabbit IgG was used alone, as control. Nuclei were stained using Hoechst 33258 (Sigma) at a final concentration of 1 μg/ml. Cytoskeleton analysis in MDA-MB231 was done using Texas red–labeled phalloidin (Sigma-Aldrich), as reported^19,20^. Fields were analyzed with a DMBL Leica fluorescence microscope equipped with HCX PL Fluotar 100 × oil objectives. Images, which are representative of three different experiments, were captured using a DC480 camera (Leica) and acquired with Application Suite (Leica) software, as reported^44,46^.

### Preparation of cytoplasmic and nuclear fractions

Cells were collected with a cellular scraper, washed with PBS, suspended in cold Harvest buffer (10 mM HEPES, pH 7.9, 50 mM NaCl, 0.5 M Sucrose, 0.1 mM EDTA, 0.5% Triton X-100) containing 1 mM sodium orthovanadate, 1 mM DTT, 100 mM NaF, 17.5 mM β-glycerophosphate, 1 mM PMSF, 4 μg/ml aprotinin as well as protease inhibitor cocktail (LAP), then incubated on ice. After 5 min, homogenates were centrifuged at 2 500 rpm to collect nuclei. The supernatants were then clarified at 15 000 rpm for 15 min and cytosolic extract was immediately used or stored at −80 °C. For nuclear extraction, nuclei were washed twice with washing buffer (10 mM HEPES pH 7.9 containing 10 mM KCl, 0.1 mM EDTA, 0.1 mM EGTA), plus freshly added protease inhibitors (1 mM DTT, 1 mM PMSF, 4 μg/ml aprotinin and LAP), then incubated with nuclear lysis buffer (10 mM HEPES pH 7.9, 500 mM NaCl, 0.1 mM EDTA, 10 mM EGTA, 0,1% Nonidet P-40), containing freshly added protease inhibitors (1 mM DTT, 1 mM PMSF, 4 μg/ml aprotinin and LAP). The nuclear extracts were vortexed at 4 °C for 15 min, then centrifuged at 15000 rpm for 10 min and immediately used or stored at −80 °C.

### Lysates, immunoprecipitation and Western blot

Lysates were prepared using lysis buffer (40 mM HEPES pH 7.5, 100 mM NaCl, 1% Triton X-100, 25 mM β-glycerophosphate, 1 mM EDTA) with freshly added protease inhibitors (1 mM PMSF, 4 mg/ml aprotinin, LAP) and 1 mM sodium orthovanadate. SDS–PAGE and Western blot analysis were done as described^59^. The mouse monoclonal anti-AR (441; Santa Cruz Biotechnology), anti-ERα (HC-20; Santa Cruz Biotechnology) and anti-ERβ (Santa Cruz Biotechnology) antibodies were used to detect AR and ER (α or β), respectively. PR was detected using the mouse monoclonal anti-PR antibody (6A1; Cell Signaling). In co-immune-precipitation experiments, lysates containing 1,5 mg/ml (for MDA-MB231 cells) or 5 mg/ml (for MDA-MB453 cells) protein were used. The mouse monoclonal anti-Src antibody (Calbiochem) was used in both co-immune-precipitation experiments and Western blot analysis^39^. The rabbit polyclonal anti-p85α antibody (clone 06–195) was from Millipore. Total FAK and P-Tyr397 FAK were detected using the mouse monoclonal anti-FAK (BD Transduction Laboratories) or the mouse monoclonal anti P-Tyr397 FAK antibodies (BD Transduction Laboratories), respectively. Total Akt and P-Ser 473 Akt were detected using the rabbit polyclonal anti-Akt (Cell Signaling) or the rabbit polyclonal anti P-Ser 473 Akt antibodies (Cell Signaling). P-Tyr118 paxillin was detected using rabbit polyclonal anti-P-Y118 paxillin antibody (BD Biosciences). Mouse monoclonal anti-paxillin antibody (clone 349; BD Biosciences) was used to detect total paxillin. Mouse monoclonal anti-tubulin antibody (Sigma-Aldrich) was used to detect tubulin. The ECL system (GE Healthcare) was used to reveal immune- reactive proteins.

### Statistical analysis

All the experiments were performed in triplicate and data are presented as mean ± standard deviation. All the comparisons for the different assays were evaluated with the paired two-tailed Student’s *t*-test. We used a *p* value of ≤0.05 or ≤0,01, both indicative of statistical significance.

## Supplementary information

Supplementary Info

## Data Availability

The data generated during the current study are available from the corresponding author on reasonable request.
