# Supplementary material for: Androgens Induce Invasiveness of Triple Negative Breast Cancer Cells Through AR/Src/PI3-K Complex Assembly
Source: Sci Rep. 2019 Mar 14;9:4490. doi: 10.1038/s41598-019-41016-4 (PMC6418124; doi:10.1038/s41598-019-41016-4)
Supplement: Supplementary file 1 — Supplementary Info [file 41598_2019_41016_MOESM1_ESM.pdf]

ANDROGEN-INDUCED INVASIVENESS IN TRIPLE NEGATIVE BREAST CANCER CELLS: ROLE OF THE AR/Src/PI3-K COMPLEX.

Pia Giovannelli, Marzia Di Donato, Ferdinando Auricchio, Gabriella Castoria*, Antimo Migliaccio

SUPPLEMENTAL DATA.

Fig. 1S. R1881 induces nuclear translocation of AR in MDA-MB231 and MDA-MB453 cells.

Quiescent MDA-MB231 and MDA-MB453 cells on coverslips were used. In the upper panels, cells were unchallenged (ctrl) or challenged (R1881) with 10 nM R1881 for 30 min. Cells were then stained for AR (α-AR; green) or nuclei (Hoechst; blue) and analyzed by IF, as described in the main text (Methods section). Images were captured and shown. Merged images are presented in right panels. Images are representative of one experiment in Fig. 1**d** and 1**e**, for MDA-MB231 and MDA-MB453 cells, respectively. Bar, 20 μM. In the lower panels, cells were challenged with 10 nM R1881 for 30 min and then stained with the secondary antibody Alexa-Fluor 488 AffiniPure Goat Anti-Rabbit IgG, as control. Nuclei were stained using Hoechst 33258. Coverslips were analyzed by IF, and images were captured as described in Methods. Bar, 10 μM.

Fig. 2S. Analysis of AR subcellular localization in cytoplasmic- or nuclear-enriched fractions from MDA-MB231 cells and study of gene transcription induced by androgens in MDA-MB231 and MDA-MB453 cells.

Quiescent MDA-MB231 cells (**a)** were left untreated or treated with 10 nM R1881 for the indicated times. Cytoplasmic- or nuclear-enriched fractions were prepared as described in the main text (Methods section) and proteins were analyzed by Western blot, using the anti-AR antibody. The fractions were also analyzed for expression of α-tubulin and histones H3, as cytoplasmic and nuclear marker, respectively.

MDA-MB231 (**b**) and MDA-MB453 (**c**) cells were transfected with pSG5-hAR encoding plasmid together with ARE-luc 3416 plasmid, as described in the main text (Methods section). Control cells were transfected with the empty pSG5. Cells were made quiescent and then left untreated or treated for with R1881 (10 nM). Luciferase activity was assayed, normalized using β-galactosidase (β-gal) as internal control, and expressed as -fold induction. Means and SEMs are shown; ***n*** represents the number of experiments. The difference in ARE-luc induction between untreated cells and those challenged with 10 nM R1881 was significant (* $p < 0.05$). Insets in **b** and **c** show the corresponding lysate proteins analyzed by Western blot, using the antibodies against AR. The filters were re-probed with anti-tubulin antibody (tubulin) as loading control.

Fig. 3S. AR is required for androgen-induced AR/Src/PI3-K/Fak complex in MDA-MB231 cells.

MDA-MB231 cells were transfected with control, non-targeting siRNA (**a**-**c**), using Lipofectamine^{TM} 2000. After transfection, the cells were made quiescent for 24 h and then left unchallenged or challenged for 5 min with 10 nM R1881, in the absence or presence of the indicated compounds. Bicalutamide (Bic; at 1μM) and the S1 peptide (at 10 nM) were

added 30 min before the hormone stimulation. Cell lysates were prepared. In **a**, lysates were immune-precipitated using the anti Src antibody (Anti Src Ab) or control IgG (control Ab). Proteins in immune-complexes were analyzed by Western blot, using the antibodies against the indicated proteins. In **b** and **c**, lysates proteins were analyzed by Western blot, using the antibodies against the indicated proteins. P-Pax stands for Tyr 118-P-Paxillin, P-akt for P-Ser 473 Akt and P-Fak stands for P-Tyr397 Fak. Filters were stripped and re-probed using anti tubulin antibody, as loading control.

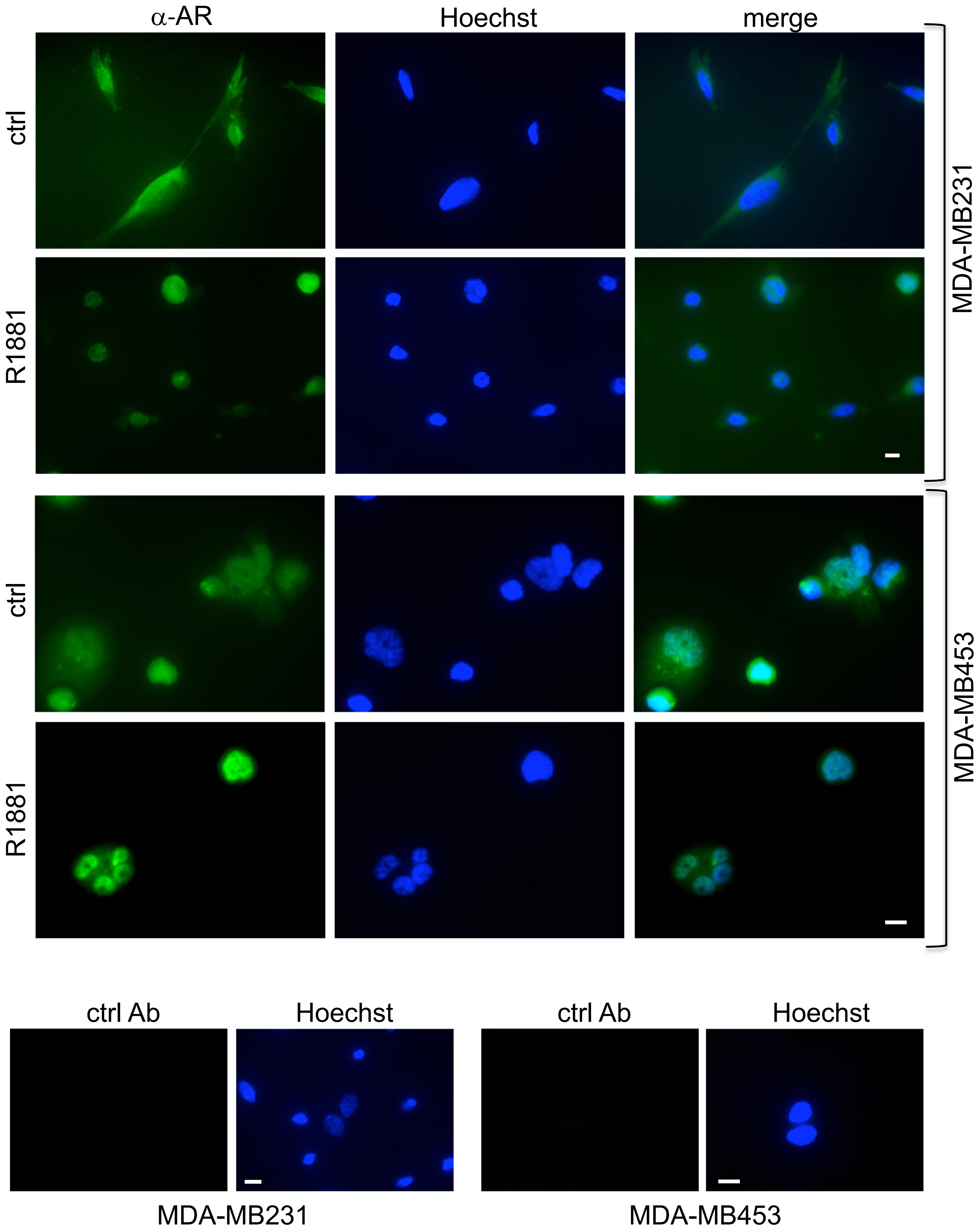
α-AR
Hoechst
merge
ctrl
R1881
ctrl
R1881
MDA-MB231
MDA-MB453
ctrl Ab
Hoechst
ctrl Ab
Hoechst
MDA-MB231
MDA-MB453

Fig. 1S

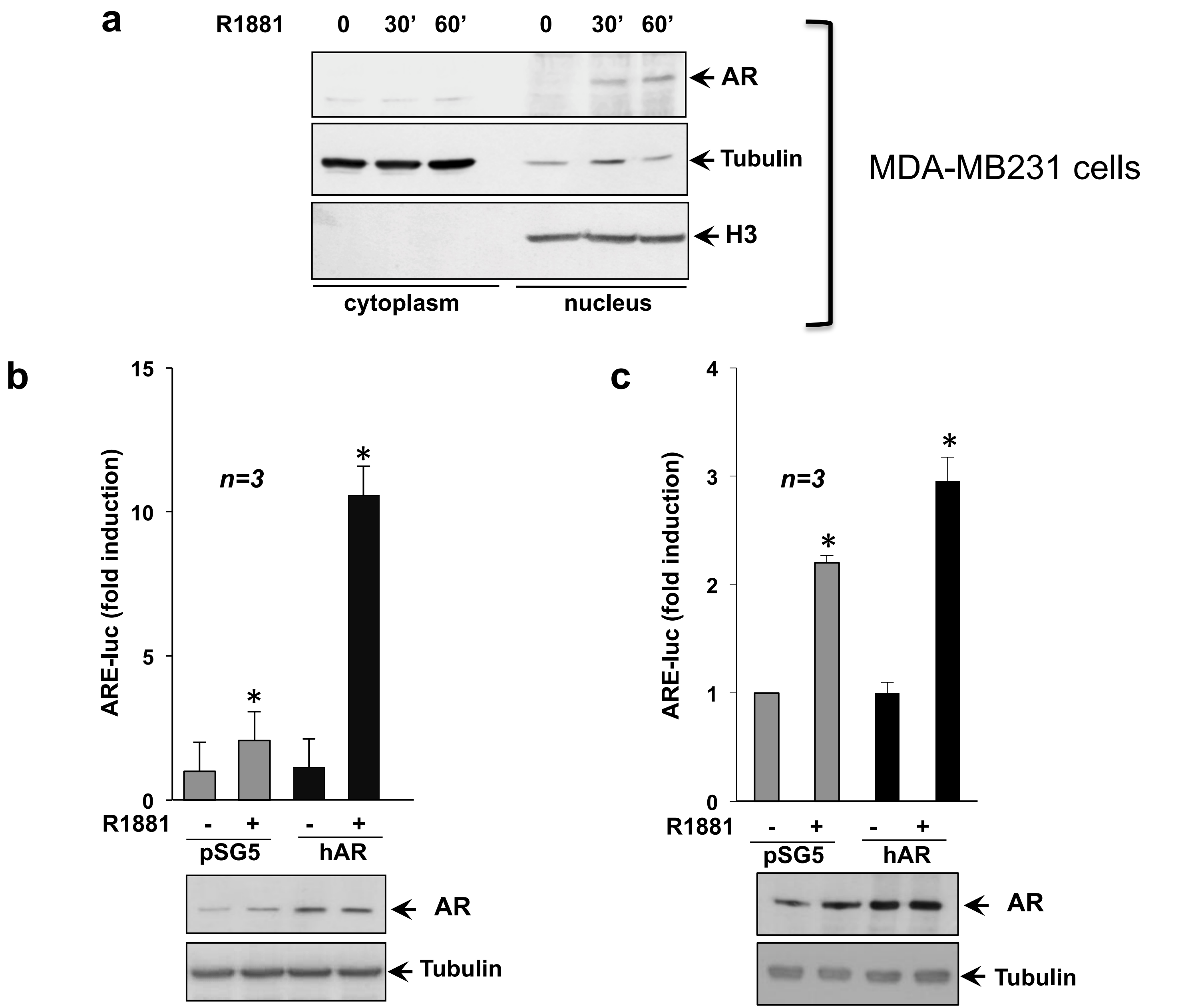
a
R1881 0 30' 60' 0 30' 60'
AR
Tubulin
H3
cytoplasm
nucleus
MDA-MB231 cells
b
15
10
5
0
ARE-luc (fold induction)
n=3
*
*
R1881 - + - +
pSG5
hAR
AR
Tubulin
c
4
3
2
1
0
ARE-luc (fold induction)
n=3
*
*
R1881 - + - +
pSG5
hAR
AR
Tubulin

Fig. 2S

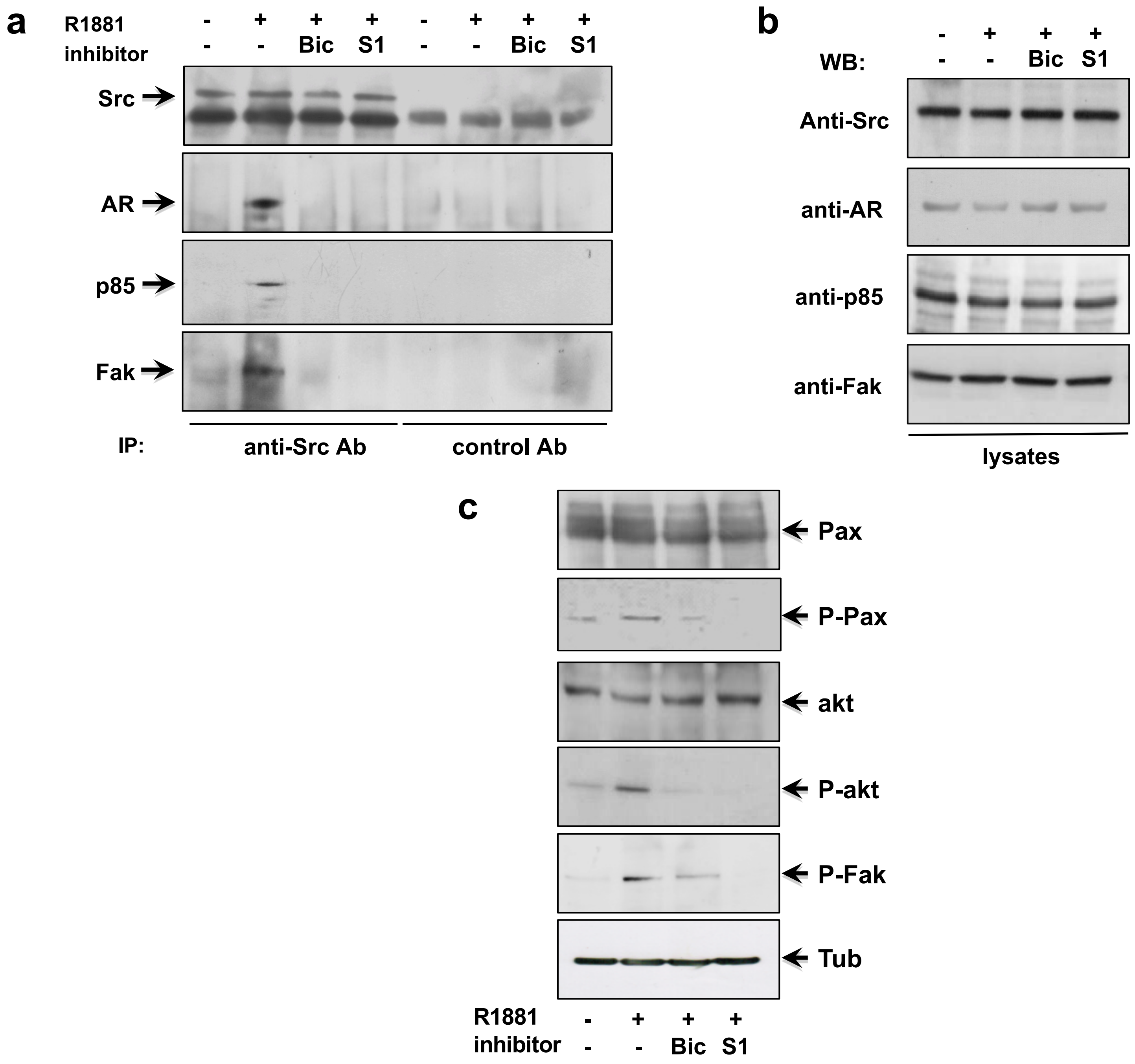
a
R1881
inhibitor
Bic
S1
Src
AR
p85
Fak
IP:
anti-Src Ab
control Ab
b
WB:
Anti-Src
anti-AR
anti-p85
anti-Fak
lysates
c
Pax
P-Pax
akt
P-akt
P-Fak
Tub

Fig. 3S
